# Evaluation and Rehabilitation after Adult Lumbar Spine Surgery

**DOI:** 10.3390/jcm13102915

**Published:** 2024-05-15

**Authors:** Tomoyoshi Sakaguchi, Sharvari Gunjotikar, Masato Tanaka, Tadashi Komatsubara, Kajetan Latka, Shashank J. Ekade, Shrinivas P. Prabhu, Kazuhiko Takamatsu, Yosuke Yasuda, Masami Nakagawa

**Affiliations:** 1 Department of Rehabilitation, Okayama Rosai Hospital, 1-10-25 Chikkomidorimachi, Minami Ward Okayama, Okayama 702-8055, Japan; tomoyoshi0127@gmail.com (T.S.); kazuhikopt0803@gmail.com (K.T.); kyushudanji19861007@gmail.com (Y.Y.); ot2632nakagawa@gmail.com (M.N.); 2 Department of Orthopedic Surgery, Okayama Rosai Hospital, 1-10-25 Chikkomidorimachi, Minami Ward Okayama, Okayama 702-8055, Japan; sharvarigunjotikar@gmail.com (S.G.); t.komatsubara1982@gmail.com (T.K.); kajto.xu@gmail.com (K.L.); drshashankjekade@gmail.com (S.J.E.); shriniprabhu31@gmail.com (S.P.P.)

**Keywords:** lumbar surgery, rehabilitation, physiotherapy, muscle exercise

## Abstract

**Purpose:** With an increase in the proportion of elderly patients, the global burden of spinal disease is on the rise. This is gradually expected to increase the number of surgical procedures all over the world in the near future. As we know, rehabilitation following spine surgery is critical for optimal recovery. However, the current literature lacks consensus regarding the appropriate post-operative rehabilitation protocol. The purpose of this review is to evaluate the optimal protocol for rehabilitation after lumbar spine surgery in adults. **Materials and Methods:** The goals of rehabilitation after lumbar spine surgery are to improve physical and psychosocial function and may include multiple modalities such as physical therapy, cognitive behavioral therapy, specialized instruments, and instructions to be followed during activities of daily living. In recent years, not only are a greater number of spine surgeries being performed, but various different techniques of lumbar spine surgery and spinal fusion have also emerged. (1) Our review summarizes post-operative rehabilitation under the following headings—1. Historical aspects, 2. Subjective functional outcomes, and (3) Actual rehabilitation measures, including balance. **Results:** Physical therapy programs need to be patient-specific and surgery-specific, such that they consider patient-reported outcome measures and take into consideration the technique of spinal fusion used and the muscle groups involved in these surgeries. By doing so, it is possible to assess the level of functional impairment and then specifically target the strengthening of those muscle groups affected by surgery whilst also improving impaired balance and allowing a return to daily activities. **Conclusions:** Rehabilitation is a multi-faceted journey to restore mobility, function, and quality of life. The current rehabilitation practice focuses on muscle strengthening, but the importance of spinal balance is less elaborated. We thus equally emphasize muscle strengthening and balance improvement post-lumbar spine surgery.

## 1. Introduction

Lower back pain is a major cause of morbidity among middle-aged and elderly individuals due to a number of possible etiologies. Even though most of the episodes of low back pain are often self-limiting, the incidence of lifetime recurrence is as high as 85% [1]. Chronic low back pain not only impairs physical and psychological health but also leads to a decline in social responsibilities, including work performance and family life, and is a major cause of increasing health care costs [2]. With advancements in medical care and the increased life expectancy of the aging population, the global burden of spinal disease has increased [3]. With the availability of advanced techniques such as minimally invasive spine surgery, percutaneous pedicle screw fixation, imaging, and navigation, a larger number of spinal surgeries are being performed currently, with some studies documenting the number of spine surgeries to be 2.4 times that of those performed 15 years ago [4,5].

Post-lumbar spinal surgery, post-operative physiotherapy intervention is crucial and recommended for improvement of post-operative functional outcome so that patients can perform their activities of daily living (ADL) at the earliest and return to normal or near normal life in the long term [6,7]. A physiotherapy regimen is supervised or home exercises with proper guidance and instruction given by a physical therapist. Furthermore, active rehabilitation is effective and important for improving short-term and long-term functional status [8]. Rehabilitation includes multiple different modalities based on the requirements of the patients, such as providing instructions, exercise therapy such as stretching and muscle strengthening, manipulation techniques, mobilization techniques, and the use of assistive equipment such as walking aids [9]. When assessing the progress of post-operative patients undergoing rehabilitation, physical therapists and surgeons often have to use disease-specific patient-reported outcome measures and standard physical performance tests. These assessments may provide useful information regarding the progress made by the patients following surgery. With different techniques of lumbar spine surgery and spinal fusion being performed, the physical therapy prescribed should be curated taking into account the technique used and should aim to target strengthening of muscle groups violated during the surgical procedure.

The benefits of physical therapy as per literature in the past have been limited to weak evidence, and the mechanisms of these benefits remain uncertain [10,11]. However, recently there have been several new reports that support the idea that rehabilitation helps to improve clinical outcomes in lumbar fusion surgery [12,13].

This article aims to summarize the historical review of rehabilitation, popular patient-reported outcome assessment methods, contemporary views on post-operative spinal rehabilitation, and ways to introduce rehabilitation after lumbar spine surgery.

## 2. Historical Review of Rehabilitation (Table 1)

The Roman army probably provided the first rehabilitation services to return wounded soldiers to work. The word “Rehabilitation” was first used in the Oxford English Dictionary in 1533. However, rehabilitation was used extensively in healthcare by 1918. After World War I, society recognized that rehabilitation was a crucial addition to services for injured or disabled patients [14]. Rehabilitation can be considered a planned and systematic societal support process offered to patients after injury or illness. Initially, orthopedic surgeons were mainly involved. The rehabilitation services that did develop in the twentieth century were initially focused on men of working age who were injured in war. Because of the increase in motorcycle accidents and sports injuries, attention has moved to people with spinal cord injuries. Spinal cord injury rehabilitation developed in the 1940s as evidence of rehabilitation’s revolutionary effectiveness [15]. After the World Health Organization (WHO) was established in 1948, they used the biopsychosocial model as a rehabilitation framework in 1980 [16].

**Figure 1:**
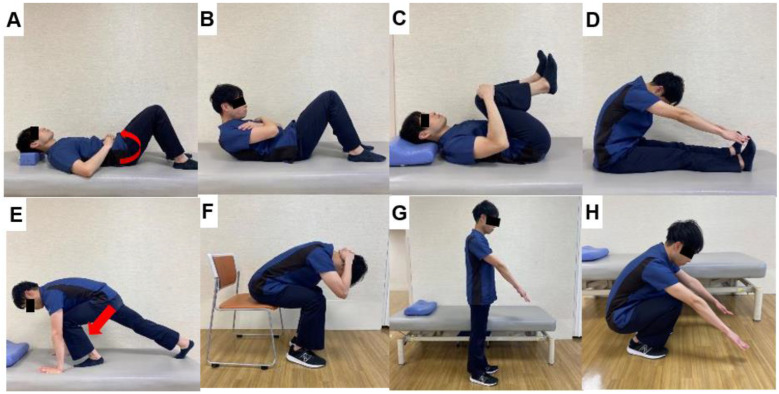
Williams lumbar flexion exercises, (**A**): Pelvic tilt (**B**): Sit-up in knee flexion (**C**): Double knees to chest to stretch the elector spine, (**D**): Seated reach to toes stretches the hamstrings and elector spine, (**E**): Forward crouch to stretch iliofemoral ligament (**F**): Seated flexion (**G**,**H**): Strengthening of quadriceps muscles and stretching of gluteus maximus and elector spine.

For lower back pain rehabilitation, lumbar stabilization exercises have become popular over the last 40 years. These exercises are focused on strengthening the muscles of truck [17]. Wiliams reported specific exercises known as Williams lumbar flexion exercises in 1937 [18] (Figure 1). These exercises are a series of therapeutic movements and stretches designed to strengthen the abdominal muscles and relax the paraspinal lumbar muscles. In 1955, Kelly addressed the importance of lumbar muscle relaxation with hanging, which is effective for lumbar foraminal enlargement, reducing muscle spasms, and facet joint release [19]. Pleasant developed and mixed Wiliams and Kelly exercises [18]. His methods consisted of three concepts: joint mobilization, soft tissue stretching, and muscle building. Calliet reported that exercise therapy positively improved blood flow and gradually strengthened the ligaments, tendons, and joint capsule, thereby aiding in the recovery of injured regions [20]. He also emphasized that resistance training enhances muscle function by increasing the cross-sectional areas of muscles, thereby preventing injury and mitigating pain further.

**Table 1:** History of important lumbar exercises.

Year	Author	Rehabilitation Method
1937	Williams [18]	Lumbar flexion exercises
1955	Kelly [19]	Hanging exercises
1962	Pheasant [20]	Posture building
1968	Calliet [21]	Lumbar lateral flexion exercises
1971	Böhler [22]	Lumbar extension exercises
1979	McKenzie [23]	Lumbar extension exercises

**Figure 2:**
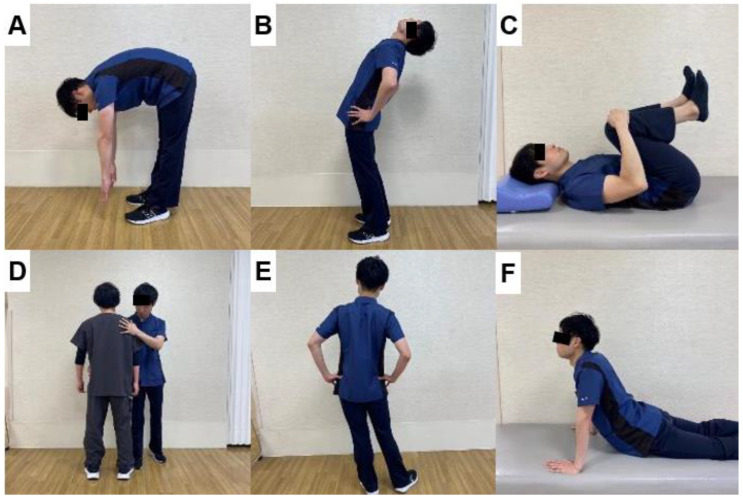
McKenzie exercises, (**A**): Flexion in standing (**B**): Extension in standing (**C**): Flexion in lying, (**D**): Therapist-assisted side glide in standing (**E**): Side glide in standing (**F**): Extension in lying.

Compared with Williams lumbar flexion exercises, Böhler emphasized the importance of lumbar extensor muscle exercises in 1971 [22]. Then, McKenzie recommended that extension exercises would reduce low backache in certain patients [23]. McKenzie exercises improve spinal mobility and promote good posture (Figure 2). Thus resulting in controlled back pain over a long duration. Recently, motor control stabilization exercises have become popular for patients with chronic, nonspecific lower back pain [24]. These exercises involve voluntary isometric contraction of the core and back muscles in a neutral spine position. WHO has released its first-ever guidelines on managing chronic low back pain in 2023 [25]. According to this guideline, a structured exercise therapy or program and spinal manipulative therapy are suggested for patients with primary chronic lower back pain.

## 3. Various Kinds of Rehabilitation

Postsurgical rehabilitation is focused on improving function through precise diagnosis, customized treatment protocols, mitigation of complications, and compensating impairment. Furthermore, rehabilitations restore and compensate for loss of functioning and prevent or deterioration in functioning in every area of a patient’s life [26]. Rehabilitation may also comprise assistive modalities, equipment, or products used to maintain, or improve function [26]. Post-surgical rehabilitation can be advised by physical therapists, occupational therapists, chiropractors, general practitioners, and orthopedic surgeons accordingly. Examples of postsurgical rehabilitation interventions are shown in Table 2.

## 4. Patients-Reported Outcome (PRO) Measures Used after Lumbar Surgery

**Figure 3:**
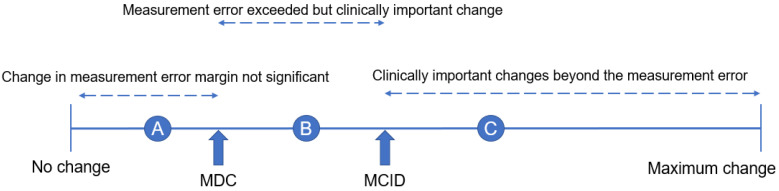
Interpretation of changes in post-treatment evaluation results, A: Post-treatment evaluation results are measurement error and clinically not important, B: The post-treatment assessment results showed changes beyond the measurement error, but not clinically important changes, C: The results of the post-treatment evaluation show clinically important changes.

PRO is useful to evaluate the various symptoms of spinal disease separately. It is possible to accurately assess the disability caused by the disease by including the impact of the spinal disease on daily life. It is necessary to use PRO to assess the impact on physical function, ADL, and quality of life. (QOL) These patient-reported outcome questionnaires are frequently used by spine surgeons and physical therapists to assess the functional outcome of patients following spinal surgery. Jaeschke et al. coined the term minimal clinically important difference (MCID) (Figure 3) [30]. The minimal detectable change (MDC) was estimated by means of the standard error of measurement in patients whose self-assessment was unchanged. The MCID describes the smallest clinical difference a patient can perceive in a specific questionnaire of data study.

Acknowledging the relevance of such an approach, additional clinically oriented concepts have been introduced that can be used to better interpret PRO measure data. The MCID is relative to the initial symptomatic state before treatment. A helpful concept to rate a cohort’s condition in absolute terms is the patient-acceptable symptom state, defined as the value on a PRO scale beyond which patients with a specific condition consider themselves well or in a satisfactory state [31]. Using all these parameters in the interpretation of evaluation outcomes, a better and patient-oriented description of the obtained success rates in therapeutic approaches can be provided. A systematic review of post-operative MCID for lumbar spine disease has been reported by Issa et al. [32] The reported MCID after surgery for lumbar spine disease is shown (Table 3).

### 4.1. Roland-Morris Disability Questionnaire (RMDQ) (Appendix A)

The RMDQ is the most commonly used lumber spine-specific assessment method [39]. Problems with the RMDQ include the lack of questions related to mental health and the fact that it is difficult for patients with only leg pain to answer [40]. The RMDQ Cronbach’s alpha values for lower back pain patients was reported 0.92 [41].

### 4.2. Oswestry Disability Index (ODI) (Appendix B)

The ODI was initially published by Fairbank to measure disability due to back pain in daily living [42,43]. Score 0–4; No disability, 5–14; Mild disability, 15–24; Moderate disability, 25–34; Severe disability, 35–50; Complete disability. ODI can evaluate ADL impairment due to lower back pain and the influence of lower limb pain, and it is correlated with lower limb pain before and after surgery [44]. ODI is more sensitive to change as compared to other general health measures when tracking the effectiveness of treatments [45]. The ODI Cronbach’s alpha values for ASD patients were reported at 0.87 [46].

### 4.3. Zurich Claudication Questionnaire (ZCQ)

The ZCQ is a disease-specific assessment of lumbar spinal stenosis (LSS) and is assessed in three domains: symptom severity, functional impairment, and treatment satisfaction [47]. The ZCQ demonstrates reliability and validity in patients with LSS and is recommended as one of the appropriate methods for evaluating LSS treatment outcomes [48]. The ZCQ Cronbach’s alpha values for lumbar canal stenosis patients was reported 0.78 [49].

### 4.4. Scoliosis Research Society 22-Item Questionnaire (SRS-22)

The SRS-22 is used to assess QOL and surgical outcomes in different types of spinal deformities [50,51]. It consists of 22 questions covering four aspects: (1) pain, (2) functioning, (3) self-image, and (4) satisfaction with the surgery [52,53]. The SRS-22 has been extensively studied and used as a reliable tool suggesting sagittal vertical axis (a marker of sagittal balance), has a significant correlation with all SRS domains, and pelvic tilt, which describes the orientation of the pelvis in relation to the body, has demonstrated correlation with SRS-22, function, and self-image domains [54,55]. The SRS-22 Cronbach’s alpha values for adolescent idiopathic scoliosis patients were reported at 0.82 [56].

### 4.5. Lumbar Stiffness Disability Index (LSDI)

The LSDI was designed and used as a tool to assess the functional impacts of lumbar spine stiffness on flexibility [57,58,59]. It is particularly used to evaluate patients after spinal fusion surgery, and it has been shown that LSDI worsens and post-operative satisfaction decreases in surgeries that involve a long fusion [60,61]. The SRS-22 Cronbach’s alpha values for lumbar fusion patients were reported at 0.89 [58].

## 5. Physical Performance Tests

The prevalence of lumbar canal stenosis increases with age and is the most common diagnosis in patients over 65 undergoing spinal surgery [62,63,64,65]. Older patients with lumbar spine disease have locomotive syndrome and reduced physical function [65,66,67]. Therefore, it is important, especially in the rehabilitation field, to assess physical function to aid in the planning of a program of therapeutic interventions. A minimal clinically important difference (MCID) has been reported in physical function assessment as well as in PRO. In general, the following assessments of physical function are used.

### 5.1. Walk Velocity (Figure 4)

Walk velocity is used to assess walking speed in meters per second over a short distance [68]. A decrease in walking speed is defined as a walking velocity of 0.8 m/s or less [69]. Changes in post-operative pain after lumbar spine surgery are associated with gait speed. Gait velocity is useful in the assessment of post-operative pain and disability after lumbar spine surgery [70]. The MCID of walk velocity after ASD surgery is 0.1 m/s [71].

**Figure 4:**
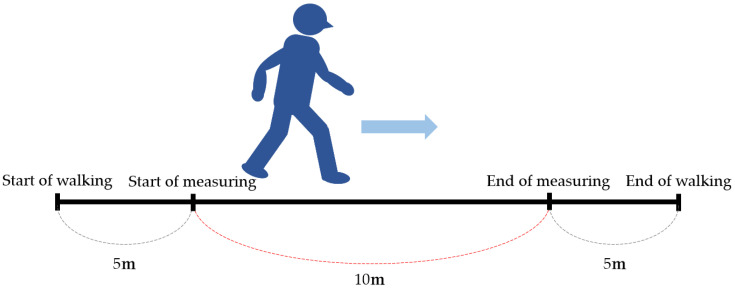
Walk velocity.

### 5.2. Six-Minute Walk Test (Figure 5)

The six-minute walk test involves walking for 6 min on a 30 m walking path and measuring the distance [72]. Six-minute walking distance is used to evaluate walking efficiency in patients with neurogenic claudication in LSS and ASD [73,74]. Self-reported walking distance in LSS patients underestimates measured walking distance by 31% and has low validity [75]. Therefore, when comparing the improvement of intermittent claudication after treatment, it is desirable to evaluate the actual walking distance using the 6 min walk test. MCID of 6 min walk distance after LSS surgery is 57.5 m [76].

**Figure 5:**
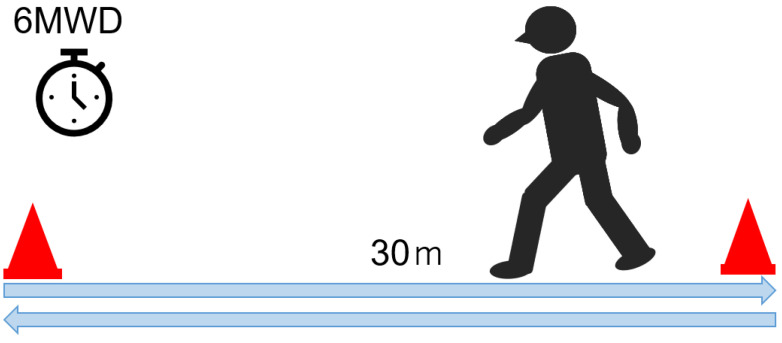
6-min walk test.

### 5.3. Timed up and Go Test (TUG) (Figure 6)

The timed up and go test (TUG) is an objective measure of functional disability that can be used to evaluate various activities such as standing, accelerating, walking, decelerating, and turning, which are often limited in patients with lumbar degenerative diseases [77]. TUG can be easily conducted with a chair and a 3 m walking space and does not require special equipment [78]. Previously TUG was used to measure motor impairment in patients with lumbar degenerative diseases, with <11.5 s classified as no impairment, 11.5 to 13.4 s as mild impairment, 13.4 to 18.4 s as moderate impairment, and >18.4 s as severe impairment [77]. TUG is not easily affected by the patient’s mental state, lifestyle, or physique [79,80] and is highly related to factors like lower limb muscle strength, sense of balance, walking ability, and risk of fall [72]. Furthermore, the TUG is used to evaluate motor function in healthy patients as well as with lumbar degenerative diseases [80,81]. Therefore, TUG is useful for evaluating dynamic balance in lumbar spine diseases. The MCID of TUG after ASD surgery and lumbar degenerative disease surgery is reported to be 2.0 s [71] and 2.1 s [82], respectively.

**Figure 6:**
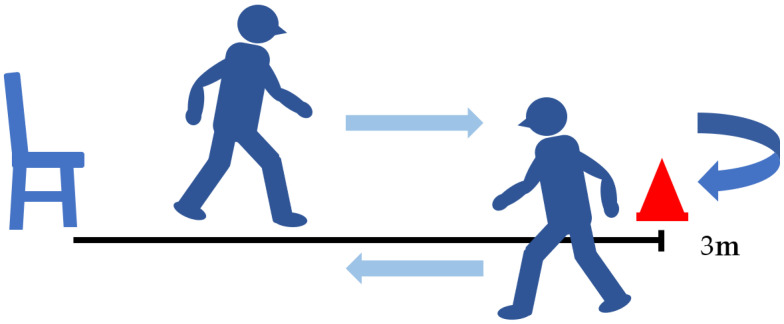
Timed up and go test.

### 5.4. Functional Reach Test (FRT) (Figure 7)

The FRT quantifies participants’ dynamic in-place standing balance control to reach distance. The distance between the starting and maximal forward reach distance beyond the participant’s arm length represents the reach distance and is recorded in centimeters [83]. Spinal mobility has been shown to significantly impact distance reached [84]. Performance of the FRT involves trunk control and depends on core and back muscle strength [85,86]. Injury to paraspinal muscles and changes in proprioception of paraspinal muscles due to lumbar spine surgery affect trunk muscle strength, leading to decreased trunk control and postural instability [87], so balance assessment using FRT is necessary.

**Figure 7:**
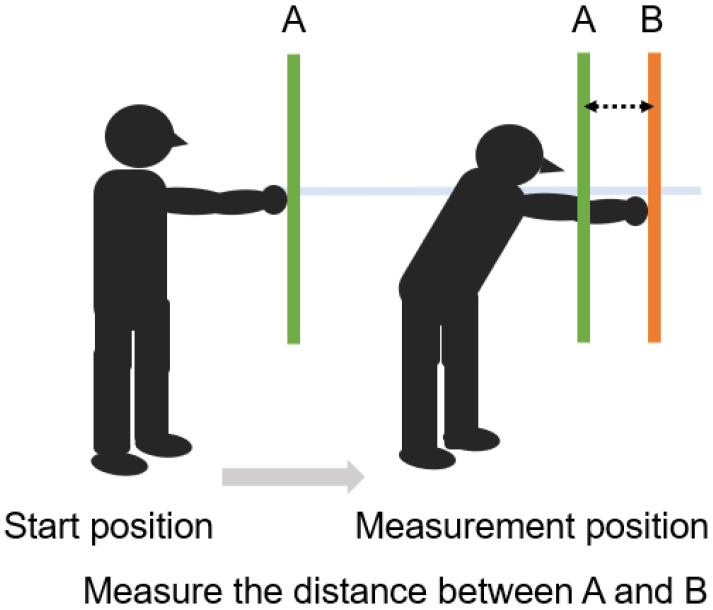
Functional reach test.

### 5.5. The Balance Evaluation Systems Test (BESTest) (Table 4)

The BESTest is a functionality scale developed to assess balance and risk of falls in the elderly [88]. It consists of 36 items and is grouped into six subsections, which represent different systems that may constrain balance, namely A: biomechanical constraints, B: stability limits/verticality, C: anticipatory postural adjustments, D: postural responses, E: sensory orientation, and F: stability in gait. Each item is scored on a four-point ordinal scale from 0 (worst performance) to 3 (best performance). Total and subscale scores are translated to a percentage score. The BESTest influences QOL in ASD [89], and the reliability of the BESTest has been reported [90]. BESTest is difficult to use in clinical practice due to its complexity, so a shortened version called Mini-BESTest [91] has been developed (Table 5).

**Table 4:** BESTest.

I. Biomechanical Constraints	II. Stability Limits	III. Anticipatory Postural Adjustments	IV. Postural Responses	V. Sensory Orientation	VI. Stability in Gait
1. Base of support	6. Sitting verticality (left and right) and lateral lean	9. Sit to stand	14. In-place response, forward	19. Sensory integration for balance, Stance on firm surface,	21. Gait level surface
2. CoM alignment	7. Functional reach forward	10. Rise to toes	15. In-place response, backward	20. Incline, EC	22. Change in gait speed
3. Ankle strength and ROM	8. Functional reach lateral	11. Stand on one leg	16. Compensatory stepping correction, forward		23. Walk with head turns, horizontal
4. Hip/trunk lateral strength		12. Alternate stair touching	17. Compensatory stepping correction, backward		24. Walk with pivot turns
5. Sit on floor and stand up		13. Standing arm raise	18. Compensatory stepping correction, lateral		25. Step over obstacles
					26. Timed “Get Up and Go” Test
					27. Timed “Get Up and Go” Test with dual task

CoM = center of mass, ROM = range of motion, CTSIB = Clinical Test of Sensory Integration for Balance, EO = eyes open, EC = eyes closed.

**Table 5:** Mini Balance Evaluation Systems Test (Mini BESTest).

Anticipatory Postural Adjustments	Postural Responses	Sensory Orientation	Dynamic Gait
1. Sit to stand	4. Compensatory stepping correction, forward	7. Stance on firm surface, EO	11. Change in gait speed
2. Rise to toes	5. Compensatory stepping correction, backward	9. Stance on foam, EC	12. Walk with head turns, horizontal
3. Stand on one leg (left and right)	6. Compensatory stepping correction, lateral (left and right)	10. Incline, EC	13. Walk with pivot turns
			12. Step over obstacles
			14. Cognitive Get up and Go

EO = Eyes Open; EC = Eyes Closed.

### 5.6. Three-Dimensional Motion Analyzers and Force Plate

Usually, gait analysis is generally performed with 3D motion analyzers [92,93] and force plates [94,95]. These devices can be used to analyze gait patterns, detailed joint movements, and gravity lines [96,97]. However, the disadvantages of these methods are cost-effectiveness, complexity of equipment operation and analysis process.

### 5.7. Triaxial Accelerometer (Figure 8)

Gait sway evaluation using an accelerometer (wearable sensor) has become a popular gait evaluation method due to its cost-effectiveness [98,99,100]. Accelerometers are easy to wear and have no limitations on measurement location, making them simple and practical tools in clinical practice [101]. Root mean square (RMS) of trunk acceleration is an indicator used to study gait sway with accelerometers [102]. RMS represents the degree of amplitude of the waveform, and a larger trunk acceleration RMS during gait indicates a greater gait sway.

**Figure 8:**
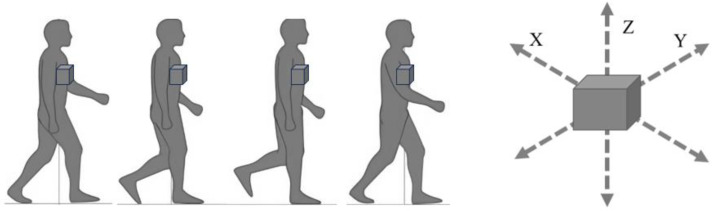
The principle of accelerometer.

## 6. Physical Therapy after Lumbar Spine Surgery

We believe that post-operative physical therapy after lumbar spine surgery is important to strengthen the affected muscles, improve balance, and facilitate return to ADL. Spinal surgery mainly includes decompression and fusion, with good post-operative results regardless of the surgical technique, and in recent years, multi-level fusion has become increasingly common [64,103]. In a comparison of surgical techniques, fixation as compared to decompression has more blood loss, operative time, and length of hospital stay [104,105].

In recent years, lateral lumbar interbody fusion (LLIF) has become more popular, with extreme lateral interbody fusion (XLIF) and oblique lumbar interbody fusion (OLIF) being the most common LLIF techniques. These techniques are less invasive than conventional posterior lumbar interbody fusion (PLIF) [106] and transforaminal lumber interbody fusion (TLIF) [107] and allows for the insertion of a larger cage, which allows for a greater restoration of lumbar lordosis [108,109].

**Figure 9:**
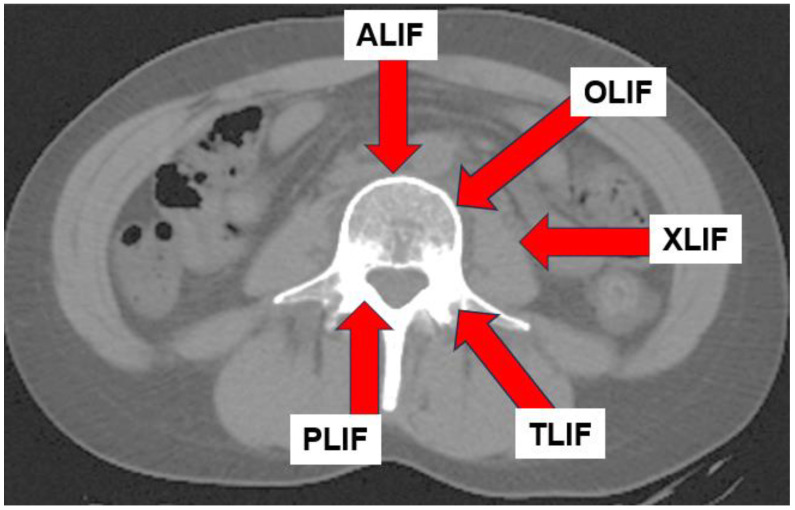
The path of entry for different spinal fusion techniques.

**Figure 10:**
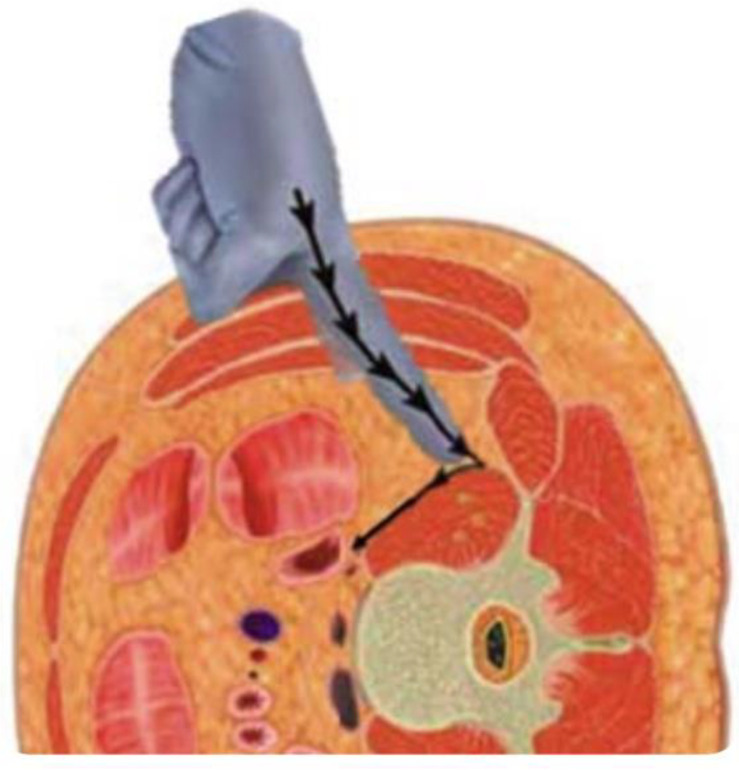
Oblique lumbar interbody fusion (OLIF) approach.

In physical therapy, it is necessary to identify the path of entry for spinal fusion and to understand the muscles involved [110] (Figure 9). Muscle atrophy results from denervation due to surgical invasion of the multifidus and erector spinae muscles for posterior approach (PLIF and TLIF) [111,112]. LLIF incises the external oblique, internal oblique, and transversus abdominis muscles, resulting in post-operative muscle weakness (Figure 10). Hence, rehabilitation should be focused according to the procedure performed, as trunk extension and trunk flexion strength strongly correlate with ODI [113].

Rehabilitation can be categorized as follows. Category 1: simple muscle power weakness, Category 2: loss of sustaining power, Category 3: spinal imbalance. The ways to proceed with the rehabilitation program after spinal surgery, considering the above stages of rehabilitation, are as below. Category 1: Exercises focused on weakness of muscles due to a surgical procedure. Category 2: Aerobic, repeated exercises for increasing the sustainability of the core, upper, and lower limbs. Category 3: Dual and multitask balance exercises improve spinal balance for improved daily activity performance.

### 6.1. Trunk Muscle Strengthening (Figure 11)

After lumbar spine fusion, motion at the level adjacent to the fusion may be altered to compensate for changes caused by the fusion, an occurrence that must also be taken into account when planning post-operative rehabilitation programs. During the early post-operative phase, strengthening exercises should be performed while keeping the lumbar spine in a neutral position to minimize strain on the fused/adjacent segment and thereafter to avoid breakage or pulling out of the implants. In functional neutral spine control exercises (NSCE), a destabilizing force acts on the trunk through loading of the extremities, and therefore proper recruitment of the trunk muscles is required to stabilize the lumbo-pelvic complex [114].

**Figure 11:**
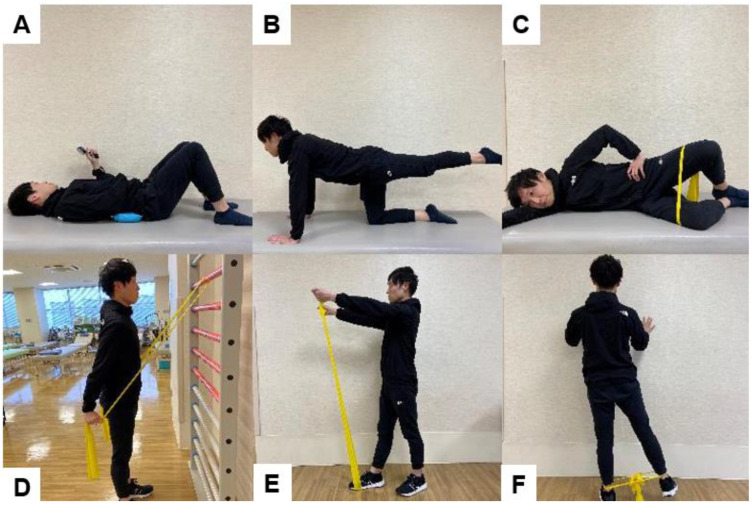
Functional neutral spine control exercises, (**A**): Drow-in [115,116], (**B**): Bird dog exercise, (**C**): Clam shell exercise, (**D**): Bilateral shoulder extension, (**E**): Bilateral shoulder flexion, (**F**): Hip abduction.

Functional NSCE mimics the trunk muscle activity patterns that occur during activities like lifting, pushing, or pulling movements [117,118]. The NSCE program has two main aims: (i) to improve control of the neutral lumbar spine and (ii) increase trunk and hip muscle coordination and strength [119]. Figure 11 shows the NSCE we have been using since the acute phase.

### 6.2. Psoas Muscle Strengthening

In XLIF, the disk space is approached through the psoas muscle. XLIF splits the psoas major muscle, resulting in muscle weakness at a rate of 9% to 31% [120]. OLIF avoids splitting of the psoas major muscle but is still associated with a 1.2% incidence of psoas muscle weakness [121]. Corrective spinal fusion for ASD with OLIF has also been shown to decrease psoas major muscle strength [122]. Strength of the psoas major muscle is related to post-operative gait sway after ASD correction [87] and to the rate of bony fusion [123], making post-operative strengthening of the psoas major muscle an important part of physical therapy programs.

**Figure 12:**
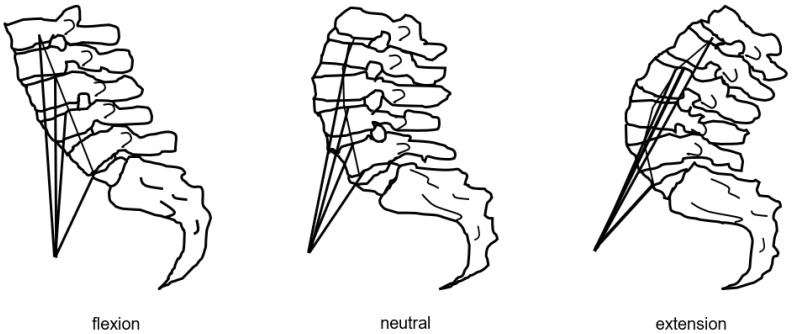
Effect of iliopsoas muscle in three positions.

**Figure 13:**
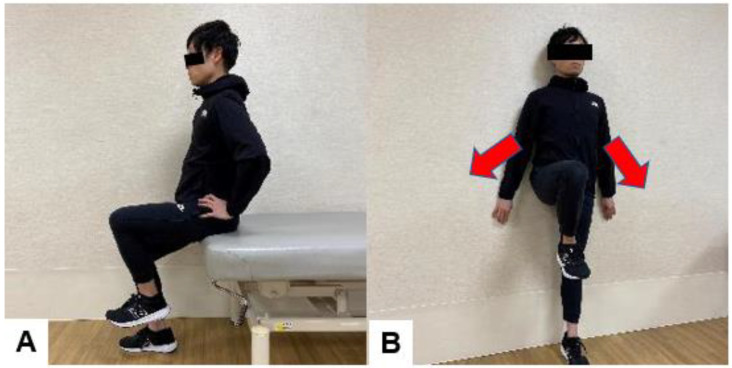
Iliopsoas muscle exercise, (**A**): Hip flexion exercise in sitting position, (**B**): Wall standing exercise.

There are some points to keep in mind when strengthening the psoas major muscle after lumbar fusion surgery. The psoas major muscle has a lumbar extension function in lumbar lordosis and a lumbar flexion function in lumbar kyphosis (Figure 12) [124]. Lumbar kyphosis is a factor in the impairment of ADL and adjacent segment diseases [125,126]. Hence, it is necessary to strengthen the psoas major muscles in a posture that can maintain the physiological lordotic position of the lumbar spine. The exercises we perform at our clinic to strengthen the psoas major muscles are shown in Figure 13.

### 6.3. Exercises to Improve Balance after Spinal Fusion Surgery

Balance dysfunction can occur after spinal surgery, increasing the risk of falls and hip fractures. Patients with long-segment thoracolumbar spine fusions had a significantly higher risk of hip fracture than those with only discectomies [127]. After a spinal fusion, ASD patients exhibit altered proprioception, sensorimotor integration failure, and postural reflex dysfunction [128]. In ASD patients after corrective spinal fusion, dynamic balance capacity improves after 6 months post-operatively [129] and is related to achieving the patient-acceptable symptom state in ODI [130]. In recent years, BESTest has been used to evaluate balance ability in ASD [84,85].

**Figure 14:**
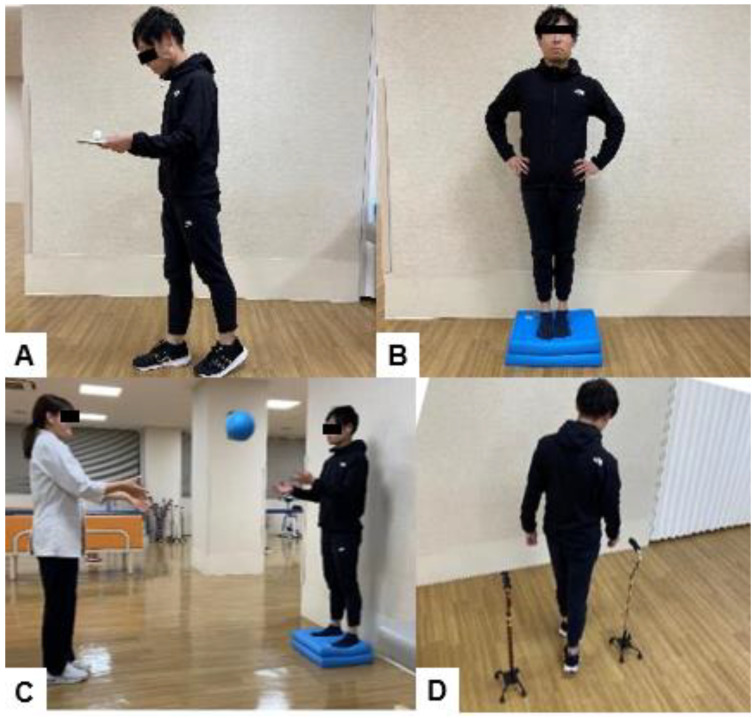
Dual- and multi-task balance exercise, (**A**): Walk while trying not to drop the ball on the tray (**B**): Stand on balance cushions with eyes closed, (**C**): Catch the ball while standing on the balance cushion, (**D**): Slalom walking with additional cognitive tasks.

It has been reported that patients with ASD have poorer BESTest results and reduced dynamic balance than healthy elderly people [84]. Halvarsson’s program includes five of the six domains of this model [131] (Figure 14). Training balance during dual-task conditions appears to be necessary to improve balance control in situations with divided attention, as balance training with single-task exercises has been shown to not transfer to dual-task performance [132].

### 6.4. Guidance on ADL after Spinal Fusion Surgery

**Figure 15:**
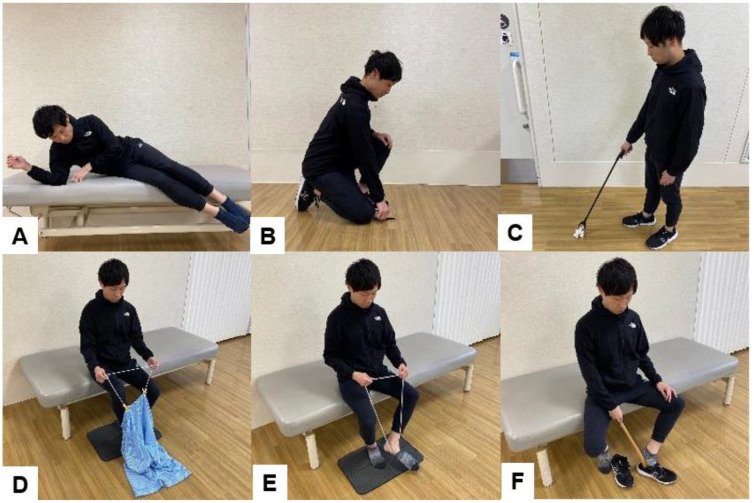
Self-help devices and coaching of ADL, (**A**): Getting up from a lateral position, (**B**): Picking up things from the floor, (**C**): How to pick up objects from the floor using self-help tools, (**D**): How to put on pants using a trouser aid, (**E**): How to put on pants using a trouser aid, (**F**): How to put on shoes using a shoehorn.

Patients who underwent a multilevel fusion, especially more than four levels, reported more limitations because of post-operative lumbar stiffness [133]. Patients with ASD after spinal corrective fusion surgery have difficulty with activities such as picking up objects from the floor, cutting toenails, maintaining personal hygiene, and putting on pants, even 2 years after surgery [134]. Lumbar spinal fusion patients with a fixed pelvis should be taught the use of self-help devices and ADL to prevent implant failure (Figure 15).

**Figure 16:**
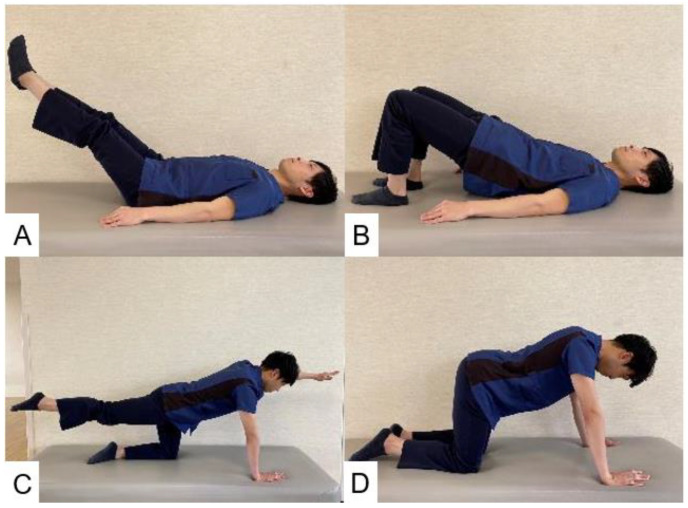
The restricted exercise, (**A**): Lifting both legs in the supine position, (**B**): Lifting of the pelvis in the supine position, (**C**): Outstretching one arm with or without simultaneously outstretching of the contralateral leg in the all-fours position, (**D**): Arching of the back in the all-fours position.

Rohlmann et al. reported movements and exercise therapy that place stress on the spine in patients undergoing lumbar corpectomy [135,136]. Movements that place stress on the spine include bending and lifting weight from the ground, forward elevation of arms with a weight in hands, tying shoes, and forward bending [135]. After lumbar spinal fusion, it is necessary to teach patients to avoid these behaviors. Exercise therapy that places stress on the spine should be avoided until bony fusion. These exercises include lifting both legs in the supine position, lifting the pelvis in the supine position, outstretching one arm with or without simultaneously outstretching contralateral leg in the all-fours position, and arching of the back in the all-fours position (Figure 16) [136]. Some exercises are not available for the elderly or fat patients. The rehabilitation personnel should select appropriate exercises for those patients.

## 7. Conclusions

Rehabilitation is a multi-faceted journey to restore mobility, function, and quality of life. Physical therapy, cognitive-behavioral therapy, and ADL are used to assess and evaluate lumbar spine disease using PROs and physical performance tests. The current rehabilitation practice focuses on muscle strengthening, but the importance of spinal balance is less elaborated. We thus equally emphasize muscle strengthening and balance improvement post-lumbar spine surgery.

## Figures and Tables

**Table 2:** Example of selected interventions for rehabilitation after lumbar surgery.

Treatment Modality	Details	Example
Patient education and self-management [9]	Teaching patient’s skills that they can use to manage their health condition	How to deal with pain The importance of physical activity in pain reduction Restrictions and working posture post-operatively (ergonomics) Mitigate pain flare-ups Step-by-step rehabilitation methods for return to routine work
Early Mobilization [27]	A subcategory of supervised or unsupervised schematic and structured exercise program (e.g., by a healthcare professional)	Stretching, Muscle strengthening Endurance exercises Neuromuscular closed chain exercises Range of motion exercise
Manual therapies [28,29]	Myofascial release: Technique that applies low-impact, prolonged stretching to the fascial complex to alleviate pain and improve function. Neural mobilization: A technique that stretches damaged nerves and improves their glide and extensibility. Manipulation: techniques incorporating a high-velocity low-amplitude impulse or thrust applied at or near the end of a joint’s passive range of motion Mobilization: techniques incorporating a low-velocity and small or large amplitude oscillatory movement, within a joint’s passive range of motion	Myofascial release Neural mobilization Massage Lumbar manipulation, mobilization
Assistive technologies	Any modalities, used to, maintain, or improve the functional capability of the patient and reduce impairment.	Walking aids Socks aids Pants aids Shoehorn Reacher

**Table 3:** MCID in PRO after surgery for lumbar spine disease.

Study	PRO	Recommended MCID	Procedure	Diagnosis
Parker [33]	ODI	14.9	TLIF	Lumber degenerative spondylolisthesis
	VAS Back	2.1		
	VAS Leg	2.8		
Parker [34]	ODI	4	Lumbar fusion	Pseudarthrosis
	VAS Back	3		
Johnsen [35]	ODI	12.88	Disk replacement	Degenerative disease
Solberg [36]	ODI	20	Discectomy	Lumbar disk herniation
	NRS Back	2.5		
	NRS Leg	3.5		
Yoshida [37]	ODI	11	Posterior corrective spinal fusion surgery	Adult spinal deformity
Fukushima [38]	ZCQ SSS	1.0	Microendoscopic laminectomy	Lumbar spinal stenosis
	ZCQ PFS	0.6		

**Figure A1:**
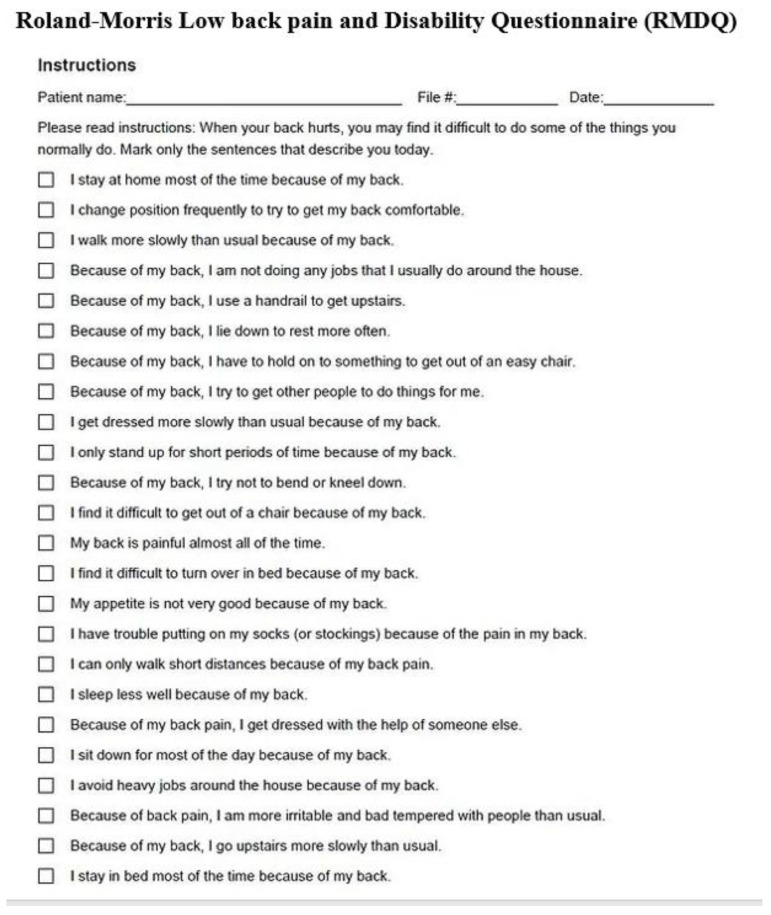
Roland-Morris Disability Questionnaire (RMDQ).

**Figure A2:**
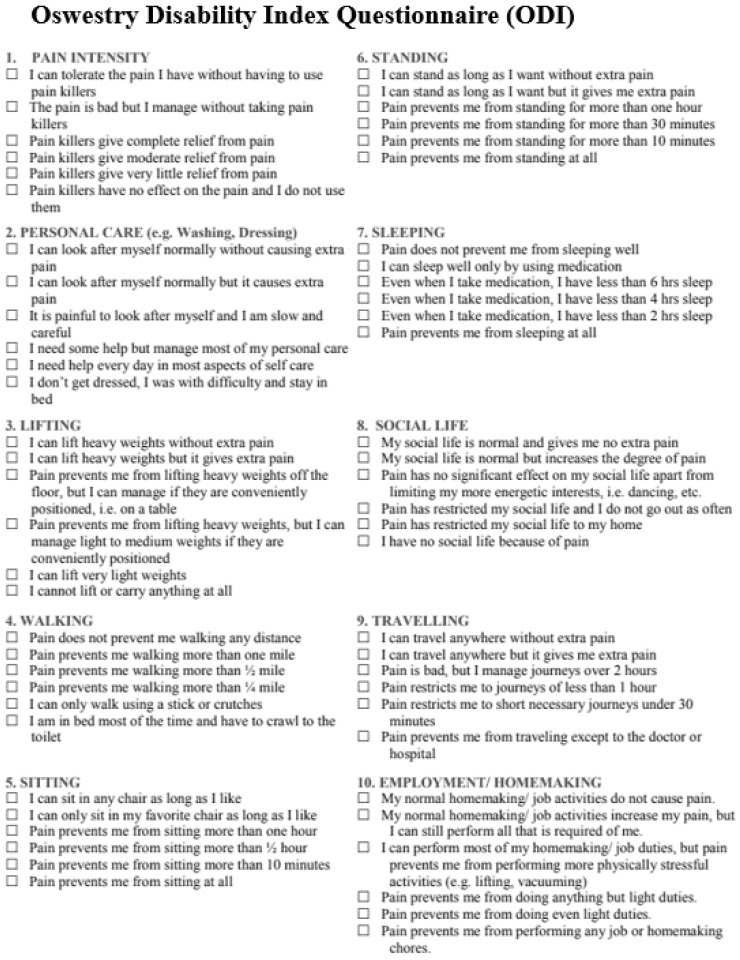
Oswestry Disability Index (ODI).

## Data Availability

The data presented in this study are available in the article.
